# Gender-specific cardiovascular outcomes in patients undergoing percutaneous coronary intervention in Chinese populations

**DOI:** 10.1186/s12872-020-01563-5

**Published:** 2020-06-09

**Authors:** Juan Long, Fanfang Zeng, Lili Wang, Chen Yi, Qiying Chen, Honglei Zhao

**Affiliations:** Department of Cardiology, Fuwai Hospital Chinese Academy Science of Medical Science, Shenzhen, China

**Keywords:** Coronary heart disease, Gender, Cardiovascular outcomes

## Abstract

**Background:**

Data was limited on the rates of in-hospital and 30-days composite outcomes between male and female patients with coronary heart disease (CHD) undergoing percutaneous coronary intervention (PCI).

**Methods:**

This was a retrospective study and CHD patients undergoing PCI between January and December of 2018 were screened and recruited. Baseline characteristics, in-hospital and 30-days composite outcomes were compared by gender. The factors influencing gender-outcome associations were evaluated.

**Results:**

A total of 672 CHD patients undergoing PCI were included into current analysis. Compared to males, females were older (53.8 ± 12.7 years vs 50.6 ± 11.8 years), more likely to be obese (32.9% vs 29.4%) and had higher prevalence of hypertension (46.7% vs 41%). Females were less likely to be smoker (30.3% vs 1.1%), have prior history of CHD (4.4% vs 10.9%), and lower socioeconomic status [SES; full-time employment (64.4% vs 71.9%), education attainment ≥ college (29.6% vs 36.8%) and annual income ≥60,000 RMB (23.7% vs 27.1%)]. Hospitalized stay was longer in females (median 5.2 vs 4.0 days), and females were more likely to experience in-hospital bleeding (3.0% vs 1.2%) and 30-days non-fatal myocardial infarction (5.9% vs 2.9%). In unadjusted model, compared to males, females had a crude odds ratio (OR) of 2.05 (95% confidence interval [CI] 1.68–2.59) for in-hospital composite outcomes and 2.16 (95% CI 1.74–2.63) for 30-days post-PCI composite outcomes. After adjustment for potential covariates, female gender remains independently associated with in-hospital and 30-days post-PCI composite outcomes. OR change was the greatest with adjustment for SES when compared to other covariates.

**Conclusion:**

Compared to male patients, female patients had a higher risk of in-hospital and 30-days composite outcomes post-PCI treatment, which were mainly attributed to the differences in SES.

## Background

Coronary heart disease (CHD) is a leading cause of morbidity and mortality in the developed countries [1–3]. In the last decades, however, the prevalence and incidence of CHD in China is increased dramatically, which is largely attributed to the endemic of cardiovascular risk factors including obesity, smoking, hypertension, dyslipidemia, and diabetes mellitus [4–6]. Notably, percutaneous coronary intervention (PCI) is one of the important therapeutic approaches for CHD patients, especially for those with acute coronary syndrome (ACS) [7]. Prior studies have demonstrated that primary PCI therapy improves the outcomes of ACS patients [8–10]. However, most of prior studies recruited patients from developed countries and female patients were always underrepresented [8–10].

China Cardiovascular Disease Report shows that the number of CHD patients undergoing PCI was increased annually in China, and compared to male patients, female patients were less likely to receive primary PCI with an ACS presentation [11–13]. Prior studies from developed countries have shown that female patients undergoing PCI had more procedure-related complications and poorer outcomes than their male counterparts, however, data on the gender-specific outcomes in Chinese CHD patients undergoing PCI were limited. With aging and endemic of cardiovascular risk factors, the prevalence and incidence of CHD among postmenopausal females were approaching to or even surpassing that of male patients [5, 14, 15]. Therefore, elucidating gender-specific outcomes among CHD patients undergoing PCI is clinically important in China.

Herein, we conducted a retrospective study and enrolled CHD patients undergoing PCI in our hospital. The aims of current study were to: 1) compare the risks of in-hospital and 30-days composite outcomes post-PCI treatment between male and female patients; 2) evaluate the association of gender and of in-hospital and 30-days composite outcomes post-PCI treatment. We believe that the results from current study would provide clues for further studies to elucidate the mechanisms underlying the gender disparities in outcomes in China, which in turn could help to reduce these disparities.

## Methods

### Study participants enrollment

**Fig. 1:**
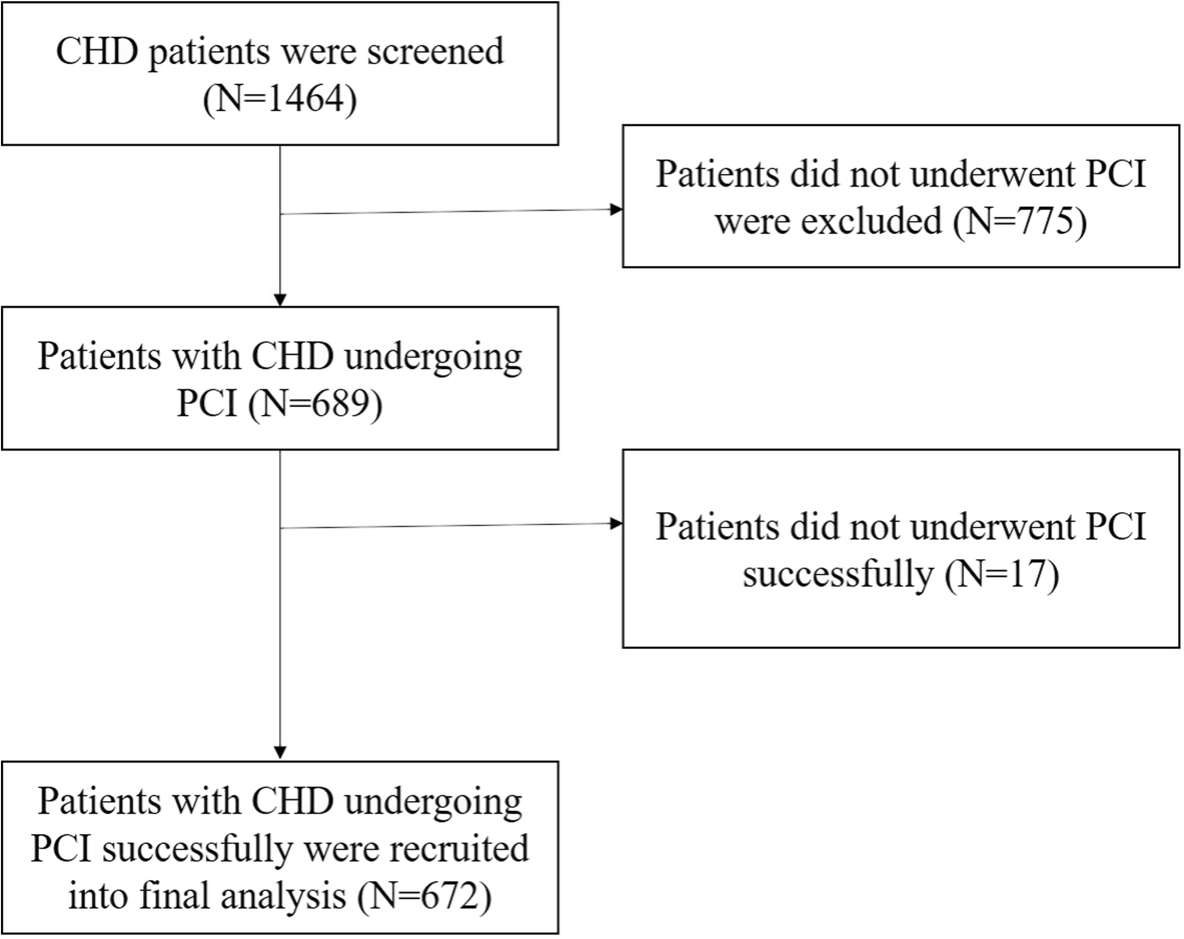
Study flow chart

This was a retrospective study and no written informed consent was required. Our current study was approved by the Clinical and Basic Research Ethic Committee of Fuwai Hospital, Shenzhen, China. From January to December of 2018, CHD patients admitted to our hospital to undergo PCI were screened, and those who did have not successful PCI were excluded. A total of 689 patients undergoing PCI were screened and 17 patients who did not underwent successful PCI were excluded (Fig. 1), and 672 patients were finally included into current analysis and were divided into male and female groups.

### Baseline characteristics collection

Baseline characteristics including demographics (age and sex), risk factors (smoking, obesity, hypertension, dyslipidemia, diabetes mellitus), comorbidities (prior history of ischemic stroke, CHD, peripheral vascular disease [PVD], chronic kidney disease [CKD], heart failure [HF]), laboratory parameters (fasting plasma glucose [FPG], total cholesterol, serum creatinine), and socioeconomic status (SES; employment status, education attainment and annual personal income) were collected from electronic health record of our hospital.

### Peri-PCI characteristics

Peri-PCI characteristics including indications for PCI (ACS or stable angina), urgent or elective PCI, radial or femoral artery access, and number and type of stents implanted were collected from electric health record of our hospital.

### Study outcomes

Study outcomes in our current analysis included in-hospital composite outcomes (acute stent thrombosis, bleeding events and all-cause mortality), and 30-days post-PCI composite outcomes (early stent thrombosis, non-fatal MI, bleeding events and all-cause mortality). Non-fatal MI was diagnosed based on clinical symptoms (e.g. chest pain), electrocardiographic changes (e.g. ST-T segment elevation or depression and T wave inversion), and presence of myocardial injury. In specific, myocardial injury was defined as plasma concentration of cardiac troponin level above the 99th percentile upper reference limit. Briefly, in our routinely clinical practice, cardiac troponin was measured using point of care first. At the same time, blood sample would be sent to the Core lab of our hospital to measure plasma concentration of cardiac troponin. Diagnosis of myocardial injury was based on plasma concentration of cardiac troponin. The Academic Research Consortium (ARC) definition was used to diagnose stent thrombosis with definite evidence from coronary angiography. Bleeding events were defined based on the Global Utilization of Streptokinase and Tissue Plasminogen Activator for Occluded Arteries (GUSTO) criteria. Medications use during hospitalization and after discharge were recorded.

### Statistical analysis

Continuous variables were presented as mean ± SD and compared by student *t* test; categorical variables were presented by number and percentages and compared by the chi-square or Fisher exact test. Logistic regression analysis was performed to evaluate the association between female gender and in-hospital and 30-days post-PCI composite outcomes (male sex was served as reference group), respectively. Statistical analyze were computed using SPSS 17.0 (SPSS Inc., Chicago, IL). All statistical tests were two-sided and considered statistically significant when *P* < 0.05.

## Results

### Baseline characteristics comparison by gender

Female patients accounted for 40.2% (*n* = 270) of current analysis. As presented in Table 1, compared to male patients, female patients were older (53.8 ± 12.7 years vs 50.6 ± 11.8 years), more likely to be obese (32.9% vs 29.4%) and had prevalence of hypertension (46.7% vs 41%). Female patients were less likely to be smoker (30.3% vs 1.1%), had prior history of CHD (4.4% vs 10.9%), and had full-time employment (64.4% vs 71.9%), education attainment ≥ college (29.6% vs 36.8%) and annual income ≥60,000 RMB (23.7% vs 27.1%). No significant between-group differences in acute coronary syndrome presentation (68.5% vs 66.9%), undergoing urgent PCI (63.7% vs 64.4%), femoral artery access (42.0% vs 43.7%), number of stents (2.1 ± 0.8 vs 2.0 ± 0.7) and drug-eluting stent implanted (96.3% vs 95.6%) were observed, except for the significant difference in length of the lesion (26.5 ± 11.4 mm vs 24.1 ± 10.3 mm).

**Table 1:** Baseline characteristics comparisons

Variables	Male (*n* = 402)	Female (*n* = 270)
Age (years)	50.6 ± 11.8	53.8 ± 12.7*
**Comorbidities**		
Obese, n (%)	118 (29.4)	88 (32.9)*
Current smoker, n (%)	122 (30.3)	3 (1.1)*
Hypertension, n (%)	165 (41)	126 (46.7)*
Diabetes mellitus, n (%)	80 (19.9)	49 (18.1)
Dyslipidemia, n (%)	124 (30.8)	84 (31.1)
Heart failure, n (%)	30 (7.7)	18 (6.7)
Coronary heart disease, n (%)	44 (10.9)	12 (4.4)*
Ischemic stroke, n (%)	23 (5.7)	14 (5.2)
Chronic kidney disease, n (%)	19 (4.7)	12 (4.4)
Peripheral vascular disease, n (%)	20 (5.0)	8 (3.0)
**Socioeconomic status**		
Full-time employment, n (%)	289 (71.9)	174 (64.4)*
Education ≥ college, n (%)	148 (36.8)	80 (29.6)*
Annual personal income ≥60,000 RMB, n (%)	109 (27.1)	64 (23.7)*
**Procedural characteristics**		
Acute coronary syndrome, n (%)	269 (66.9)	185 (68.5)
Urgent PCI status, n (%)	256 (63.7)	174 (64.4)
Femoral artery access, n (%)	169 (42.0)	118 (43.7)
Lesion length (mm)	26.5 ± 11.4	24.1 ± 10.3*
Number of stents	2.1 ± 0.8	2.0 ± 0.7
Drug-eluting stents, n (%)	387 (96.3)	258 (95.6)
**Laboratory parameters**		
Fasting plasma glucose (mg/dL)	92.6 ± 12.5	91.3 ± 13.4
Total cholesterol (mmol/L)	5.0 ± 0.9	5.0 ± 1.0
Creatinine (umol/L)	63.5 ± 21.2	60.6 ± 19.5

*PCI* Percutaneous coronary intervention; * *P* < 0.05 versus male patients

### Medications used comparison by gender

Medications used during hospitalization and after discharge were compared. As presented in Table 2, no significant differences in medications used during hospitalization and follow-up by gender were observed except for a lower use of angiotensin converting enzyme inhibitor/angiotensin receptor blocker.

**Table 2:** Medications used comparisons by gender

Variables	Male (*n* = 402)	Female (*n* = 270)
**During hospitalization**		
Aspirin, n (%)	402 (100)	270 (100)
Clopidogrel, n (%)	368 (91.5)	246 (91.1)
Ticagrelor, n (%)	32 (8.0)	20 (7.4)
Statins, n (%)	380 (94.5)	252 (93.3)
ACEI/ARB, n (%)	342 (85.1)	220 (81.5)*
Beta-blocker, n (%)	330 (82.1)	218 (80.7)
LMWH, n (%)	25 (6.2)	12 (4.4)
Warfarin, n (%)	0 (0)	0 (0)
PPI, n (%)	219 (54.5)	152 (56.3)
**During follow-up**		
Aspirin, n (%)	398 (99.0)	265 (98.1)
Clopidogrel, n (%)	362 (90.0)	242 (89.6)
Ticagrelor, n (%)	28 (7.0)	16 (5.9)
Statins, n (%)	380 (94.5)	252 (93.3)
ACEI/ARB, n (%)	340 (84.6)	215 (79.6)*
Beta-blocker, n (%)	330 (82.1)	216 (80.0)
LMWH, n (%)	0 (0)	0 (0)
Warfarin, n (%)	3 (0.7)	2 (0.7)
PPI, n (%)	187 (46.5)	132 (48.9)

*ACEI/ARB* Angiotensin converting enzyme inhibitor/angiotensin receptor blocker, *LMWH* Low molecular weight heparin, *PPI* Proton pump inhibitor; * *P* < 0.05 versus male patients

### Comparisons of outcomes

As presented in Table 3, compared to male patients, hospitalized stay was longer in female patients (median days: 5.2 vs 4.0), and female patients were more likely to experience in-hospital bleeding (3.0% vs 1.2%) and 30-days non-fatal myocardial infarction (5.9% vs 2.9%). No significant gender differences in acute- and early-stent thrombosis, 30-days bleeding and all-cause mortality were observed.

**Table 3:** Comparisons of outcomes

Outcomes	Male (*n* = 402)	Female (*n* = 270)
**In-hospital**		
Median stay in hospital (days)	4.0 (2.9–6.1)	5.2 (3.3–6.8)*
Bleeding events, n (%)	5 (1.2)	8 (3.0)*
Acute stent thrombosis, n (%)	2 (0.5)	3 (1.1)
All-cause mortality, n (%)	0 (0)	0 (0)
**30-days**		
Bleeding events, n (%)	8 (2.0)	5 (1.9)
Early stent thrombosis, n (%)	2 (0.5)	2 (0.7)
Non-fatal myocardial infarction, n (%)	2 (0.5)	7 (2.6)*
All-cause mortality	0	1 (0.4)

### Association of female gender and outcomes

As presented in Table 4, in unadjusted model, compared to male patients, female patients had a crude odds ratio of 2.05 for in-hospital composite outcomes. After adjusted for age, the odds ratio reduced by 22%; with further adjustment for comorbidities in model 2, the odds ratio reduced by 28%; and after adjustment for SES in model 3, the odds ratio reduced by 35%, with odds ratio of 1.20 (95% confidence interval 1.10–1.43).

**Table 4:** Associations of female gender and composite outcomes

Models	Odds ratio	95% Confidence interval
**In-hospital composite outcomes**		
Unadjusted	2.05	1.68–2.59
Model 1	1.83	1.50–2.22
Model 2	1.55	1.30–1.92
Model 3	1.20	1.10–1.43
**30-days composite outcomes**		
Unadjusted	2.16	1.74–2.63
Model 1	1.97	1.62–2.40
Model 2	1.60	1.35–1.94
Model 3	1.20	1.12–1.51

Model 1, adjusted for age; model 2, further adjusted for chronic kidney disease, diabetes mellitus, hypertension, dyslipidemia, and lesion length; model 3, further adjusted for annual income and education attainment

Female patients also had a crude odds of 2.16 for 30-days composite outcomes. After adjusted for age, the odds ratio reduced by 19%; with further adjustment for comorbidities, the odds ratio reduced by 37%; and after adjustment for SES, the odds ratio reduced by 40%, with odds ratio of 1.20 (95% confidence interval 1.12–1.51).

## Discussion

There are two important findings of current study. First, compared to male patients, female patients have higher risk of in-hospital bleeding and 30-days non-fatal MI. Second, after adjustment for potential covariates, female gender remains independently associated with in-hospital and 30-days composite outcomes. Further researches are needed to elucidate the underlying mechanisms and to implement interventions targeting the mechanisms for observed gender differences in clinical outcomes so as to narrow these health disparities by gender.

Numerous studies from the United States and European countires have consistently demonstrated that compared to male patients, female patients undergoing PCI have higher rates of re-hospitalization and all-cause mortality. For example, one systemic review shows that compared to males with ST-segment elevation myocardial infarction (STEMI), females with STEMI had higher mortality rate and this difference was partially attributed to the differences in baseline cardiovascular risk profiles [16]. Otten AM et al. reported that the gender differences in mortality post primary PCI were age dependent, and although young women had a lower risk before PCI, survival rate was lower in women when compared to similarly aged men [17]. In patients with ACS, Pendyala et al. found that compared to men, women experienced higher 1-year risk of mortality and major adverse cardiovascular events post-PCI [18]. One recent study from Chinese population with ACS showed that women received less optimal treatment than men during hospitalization, and the higher in-hospital mortality rate in women was majorly attributed to the difference in clinical profiles and in-hospital treatment [3]. Consistent with prior studies, our current study also demonstrates that female patients undergoing PCI has a higher risk of in-hospital and 30-days composite outcomes. These differences were mainly driven by the differences in in-hospital bleeding events and 30-days non-fatal myocardial infarction. However, it was worth to note that there were some differences between our current study and the prior study from Chinese populations [3]. First, prior Chinese study enrolled ACS patients while current study enrolled both stable and ACS patients. Second, participants in prior study had higher comorbid burdens than participants in our current study as reflected by the older age, higher prevalence of hypertension, diabetes mellitus, CKD and among others in prior study. Third, prior study only evaluated the in-hospital events while our current study evaluated both in-hospital and 30-days events after PCI treatment. Although somewhat differences, both our current study and prior large national study together suggested that women with CHD undergoing PCI had higher cardiovascular risks and sex-specific managements are needed to narrow these health disparities in China.

The mechanisms attributed to gender differences in outcomes vary between different studies, including differences in biological features, baseline cardiovascular risk profiles, treatment received during hospitalization and after discharge, medication adherence, and among others [19–22]. In current study, we used regression models to evaluate the changes of odds ratio between different models. We observed that the association of female gender and in-hospital and 30-days composite outcomes were attenuated with greatest magnitude after adjustment for SES, suggesting that compared to age and other traditional cardiovascular risk factors, SES played a more important role in gender differences in clinical outcomes. In current analysis, we included education attainment, personal annual income and employment status as indicators of SES. Numerous studies have demonstrated that compared to individuals with higher SES, those with lower SES had higher cardiovascular risk post-PCI treatment. To our knowledge, this was the first study to evaluate the influence of SES on gender differences in clinical outcomes post-PCI treatment in Chinese populations. The explanations for SES-mediated gender differences in clinical outcomes might be multifactorial such as poor lifestyle and behavior (e.g. smoking and physical inactivity), greater burden of risk factors, and lower medications adherence [23–25]. Future studies are needed to corroborate our findings as well as to elucidate the underlying mechanisms in Chinese populations. Lastly, it is important to note that prior study has shown the urban-rural disparities in treatments and outcomes after ST-elevation myocardial infarction in China [26]. However, current study did not have the ability to evaluate whether the gender differences in clinical outcomes were attributed to the differences between urban and rural resident because all participants were from the urban area of Shenzhen city and the medical costs were universal paid by the health insurance.

There are some limitations of current study. First of all, this was a retrospective study and findings from current analysis should not be drawn a causal relationship. Second, although we had adjusted for potential confounding factors, the undetected and unmeasured factors might still exist that could influence the gender-outcome association. Third, current study was conducted in Chinese populations and whether the findings can be extrapolated to other race/ethnic groups was unknown. Fourth, since we had only followed up patients for 30 days, whether the gender differences in clinical outcomes persist with longer follow-up was also unknown. Fifth, the sample size of the current analysis was relatively small and large prospective studies are needed to corroborate the current findings. Last but not the least, this was a single center retrospective study, and as mentioned above that there were some differences in clinical profiles between current study and prior national prospective cohort study from Chinese populations, future studies are needed to evaluate the reasons for these different findings across studies through collaboration.

## Conclusion

In conclusion, our current study shows that in CHD patients, compared to male patients, female patients have a higher risk of in-hospital and 30-days composite outcomes post-PCI treatment. The differences in clinical outcomes are mainly attributed to the differences in SES.

## Data Availability

The datasets used and/or analysed during the current study available from the corresponding author on reasonable request.

## Abbreviations

CHD: Coronary heart disease
MI: Myocardial infarction
PCI: Percutaneous coronary intervention
ACS: Acute coronary syndrome
PVD: Peripheral vascular disease
CKD: Chronic kidney disease
HF: Heart failure
FPG: Fasting plasma glucose
SES: Socioeconomic status
CI: Confidence interval
